# Genetic Parameter Estimation and Breeding Value Ranking for Litter Size Traits in Meat Rabbits: REML and Bayesian Inference Under Limited Data Conditions

**DOI:** 10.3390/ani16142192

**Published:** 2026-07-14

**Authors:** Fabián Magaña-Valencia, Raymundo Rodríguez-de-Lara, Rodolfo Ramírez-Valverde, Rafael Núñez-Domínguez, Jorge Hidalgo

**Affiliations:** 1 Department of Animal Science, Universidad Autonoma Chapingo, Chapingo, Texcoco 56230, State of Mexico, Mexico; fmaganav@chapingo.mx (F.M.-V.); rrodriguezd@chapingo.mx (R.R.-d.-L.); rnunezd@chapingo.mx (R.N.-D.); 2 Department of Animal and Dairy Science, University of Georgia, Athens, GA 30602, USA; jh37900@uga.edu

**Keywords:** rabbit breeding, variance components, animal model, breeding values, Gibbs sampling

## Abstract

Reproductive efficiency is a key factor in meat rabbit production; however, estimating variance components for litter size can be challenging in populations with limited data. Our objective in this study was to estimate genetic parameters for litter size in meat rabbits using frequentist and Bayesian approaches. A total of 956 kindling records were analyzed, with approximately 40–48% of does contributing only a single record. The traits analyzed were the number of kits born alive, stillborn, and total born, and litter size at 7, 35, and 70 days. Variance components were estimated using restricted maximum likelihood (REML) and Gibbs sampling as implemented in the BLUPF90 suite of programs. Univariate and bivariate mixed models were fitted, including additive genetic effects, with and without the permanent environmental effect. Heritability estimates were low for all traits (<0.10), with slightly higher estimates under the Bayesian approach. Including the permanent environmental effect improved model fit but caused convergence problems with REML, whereas the Bayesian approach yielded non-zero estimates and an explicit characterization of uncertainty through posterior distributions. Although overall correlations between predicted breeding values were high, minor changes in animal rankings were detected when comparing full and reduced models under the Bayesian approach. These results highlight the importance of inference methods and model specification in genetic evaluations based on limited-information datasets.

## Simple Summary

Estimating the genetic merit for the number of kits a doe can produce and successfully raise is essential for improving meat rabbit production efficiency. However, in some cases, limited data make it difficult to obtain reliable estimates of the animals’ genetic potential for survival and reproductive traits. In this study, kindling records from four rabbit breeds raised in Mexico were analyzed to estimate the extent to which variation in litter size and survival is due to genetic factors and to the environment. Two widely used statistical approaches in animal genetics were compared: one based on traditional mathematical calculations and another based on probability and simulation. Both approaches confirmed that litter size is mainly influenced by environmental factors. Differences between estimation methods became more evident under the limited-information conditions represented by the present dataset. In addition, slight changes in breeding value rankings were observed when alternative models were compared. These results show that the choice of both the statistical method and the model can influence selection decisions, especially when working with limited data. Improving litter size and survival contributes to more efficient and sustainable rabbit production by reducing losses and supporting the supply of high-quality animal protein for human consumption.

## 1. Introduction

Litter size is an economically important trait in meat rabbit production, as it directly influences productive efficiency and the profitability of the production system [1,2,3]. Accurate estimation of variance components and genetic parameters such as heritability, repeatability, and genetic correlations is essential for the design and evaluation of breeding programs [4,5]. However, litter size traits are statistically challenging because they are typically characterized by low heritability and substantial environmental influence [1,6,7]. Furthermore, litter size is a repeated trait recorded across successive parities, requiring the separation of additive genetic, permanent environmental, and residual sources of variation [8,9]. This partition becomes particularly difficult when the number of records per female is limited, reducing the amount of information available for variance component estimation [10].

Many rabbit populations and emerging breeding programs operate under conditions of limited information, where population size is relatively small and the number of repeated records per individual is limited [11]. Under these conditions, the estimation of variance components may be affected by numerical instability, lack of convergence and increased uncertainty. These challenges primarily arise from limitations in the amount of information available and reduced parameter identifiability rather than computational constraints [10,12].

In quantitative genetics, two statistical approaches are widely used for estimating variance components: the frequentist approach based on restricted maximum likelihood (REML) and the Bayesian approach using Markov Chain Monte Carlo with Gibbs sampling (MCMC-GS). The REML method has a solid theoretical foundation and well-established asymptotic properties, ensuring efficient estimates when the sample size is sufficiently large [13,14,15]. It is also the standard approach implemented in genetic evaluation programs widely used in animal breeding [16,17]. Nevertheless, the numerical performance of REML-based algorithms depends on model complexity, data structure and the amount of information available [18].

In contrast, the Bayesian approach based on MCMC-GS provides posterior distributions for any sample size and explicitly represents the uncertainty associated with the estimated parameters. These features may be advantageous in situations where information is limited, or parameter estimation is challenging. However, Bayesian analyses require additional decisions regarding prior specification and convergence assessment. When the sample size is large, the posterior distribution of the parameters tends to approach asymptotic normality [18,19], and both approaches are expected to yield equivalent results [14].

Although litter size traits have been extensively studied in rabbit breeding populations [1,20,21,22], less information is available regarding the performance of alternative variance component estimation methods when applied to datasets with limited information. This issue is particularly relevant for small populations and breeding programs in which the number of records per animal is restricted [14,18].

In this context, the objective of the present study was to estimate variance components and genetic parameters associated with litter size in meat rabbits using REML and MCMC-GS and to evaluate the impact of estimation method and model specification on prediction and rankings of breeding values under limited-information conditions.

## 2. Materials and Methods

### 2.1. Data Source, Animals, and Management

The data used in this study were obtained from the Applied Research Unit in Rabbit Production of the Universidad Autónoma Chapingo. Records from 440 does of the New Zealand White, Californian, Chinchilla, and Brown breeds were used. Animals were mated simultaneously and randomly within breed, avoiding matings between close relatives (full siblings, half-siblings, or parent–offspring). Does were managed in two groups with mating at 11 days postpartum. Matings were performed by natural service or artificial insemination. Pregnancy diagnosis was conducted by abdominal palpation between 12 and 14 days after mating; non-pregnant does were reassigned to the subsequent mating group. Kits were weaned at 35 days of age and slaughtered after 70 days of age. The pedigree used for genetic analyses included all available ancestry information and comprised 3481 rabbits, including 399 founders, 180 sires and 364 dams represented in the relationship matrix, and reached a maximum pedigree depth of four generations.

### 2.2. Traits Studied

For each kindling, the number of kits born alive (BA), born dead (BD), and total born (TB) was recorded, as well as litter size at 7, 35, and 70 days of age (LS7, LS35, and LS70, respectively). In total, 956 records were analyzed for BA, BD, and TB, and 819, 804, and 725 records for LS7, LS35, and LS70, respectively. The structure of the dataset, including the number of records per trait, the number of does with available information, and the distribution of parities per doe, is presented in Table 1. The reduction in the number of records across traits was mainly due to stage-specific data availability at the time of data extraction.

### 2.3. Statistical Analysis

The statistical analysis was conducted jointly for the four genetic groups, assuming common variance components across breeds. The effects included in the models for estimating (co)variance components are shown in Table 2. The fixed or systematic effects considered were breed, contemporary group (does served within a 5-day interval and housed in the same gestation or kindling barn), type of service (natural mating and artificial insemination with 0.15 or 0.20 mL of gonadotropin-releasing hormone), and the physiological status of the doe at mating (nulliparous, primiparous lactating, primiparous non-lactating, multiparous lactating, and multiparous non-lactating). The random effects included the animal’s additive genetic effect and the doe’s permanent environmental effect.

#### 2.3.1. Variance Components Estimation

Variance components were estimated using restricted maximum likelihood with the average information (AI-REML) and expectation–maximization (EM-REML) algorithms, as well as a Bayesian approach based on Markov Chain Monte Carlo with Gibbs sampling. Analyses were performed using the BLUPF90+ (version 2.56), GIBBSF90 (version 3.22), and POSTGIBBSF90 (version 3.15) software packages [16]. For REML analyses, convergence was declared when the relative change in variance component estimates between successive iterations was lower than 10^−12^, with a maximum of 10,000 iterations allowed.

##### Univariate models

The univariate animal model was:

y=Xβ+Zu+Wpe+e

where *y* is the vector of observations; *β* is the vector of systematic effects; *u* is the vector of random direct additive genetic effects; *pe* is the vector of random permanent environmental effects of the doe (defined at the individual doe level, accounting for repeated records of the same female across parities); and *e* is the vector of residual effects. *X*, *Z*, and *W* are incidence matrices relating records to *β*, *u*, and *pe*, respectively.

Random effects were assumed to be distributed as:

$$u∼N\left(0,A\sigma_{u}^{2}\right),\;\;pe∼N\left(0,I\sigma_{pe}^{2}\right),\;\;e∼N(0,I\sigma_{e}^{2})$$

where *A* is the additive genetic relationship matrix, *I* is an identity matrix, and $\sigma_{u}^{2}$, $\sigma_{pe}^{2}$, and $\sigma_{e}^{2}$ are the additive genetic, permanent environmental, and residual variance components, respectively.

Heritability ($h^{2}$) and repeatability (*re*) were estimated as:

$$h^{2}=\frac{\sigma_{u}^{2}}{\sigma_{u}^{2}+\sigma_{pe}^{2}+\sigma_{e}^{2}},\;\;re=\frac{\sigma_{u}^{2}+\sigma_{pe}^{2}}{\sigma_{u}^{2}+\sigma_{pe}^{2}+\sigma_{e}^{2}}$$

##### Bivariate Analyses

Genetic ($r_{g}$), permanent environmental ($r_{pe}$), and residual ($r_{e}$) correlations between traits were estimated using bivariate analyses based on the following model:

$$\left[y_{1}\;y_{2}\;\right]=\left[X_{1}\;0\;0\;X_{2}\;\right]\left[\beta_{1}\;\beta_{2}\;\right]+\left[Z_{1}\;0\;0\;Z_{2}\;\right]\left[u_{1}\;u_{2}\;\right]+\left[W_{1}\;0\;0\;W_{2}\;\right]\left[{pe}_{1}\;{pe}_{2}\;\right]+\left[e_{1}\;e_{2}\;\right]$$

where subscripts 1 and 2 denote the traits analyzed. Incidence matrices and vectors are defined as in the univariate model.

Random effects were assumed to follow multivariate normal distributions:

$$\left[u_{1}\;u_{2}\;\right]∼N\left(0,G⊗A\right),\;\;\left[{pe}_{1}\;{pe}_{2}\;\right]∼N\left(0,C⊗I\right),\;\;\left[e_{1}\;e_{2}\;\right]∼N(0,R⊗I)$$

where *G*, *C*, and *R* are 2 × 2 genetic, permanent environmental, and residual (co)variance matrices, respectively.

##### Bayesian Inference

Under the Bayesian framework, traits and random effects were assumed to follow conditional normal distributions:

$$\left[y_{1}\;y_{2}\;\right]|\beta ,u,pe,∼N(X\beta +Zu+Wpe,R⊗I)$$

with prior distributions:

$$u∼N\left(0,G⊗A\right),\;\;pe∼N\left(0,C⊗I\right),\;\;e∼N(0,R⊗I)$$

Variance–covariance matrices (*G*, *C*, *R*) were assumed to follow inverse Wishart prior distributions, while systematic effects were assigned a uniform prior distribution. The default GIBBSF90 prior specification was used, with degrees of freedom equal to −2 and 2 × 2 scale matrices with 1.0 in the diagonals and 0.1 in the off-diagonals.

For the final univariate analysis of variance components, posterior distributions were constructed using 200,000 samples, with a burn-in period of 10,000 samples and a thinning interval of 50, resulting in 3800 retained samples per analysis. Monte Carlo standard errors were evaluated to assess sampling precision. Convergence was assessed using the Geweke diagnostic, visual inspection of trace plots, posterior density stability, and effective sample size. All chains satisfied the commonly accepted Geweke criterion (|Z| < 2). Summary convergence diagnostics are presented in Table S8, and representative trace plots are shown in Figure S1 (Supplementary Materials). Posterior symmetry was evaluated by comparing posterior means and medians. MCMC settings used for univariate analyses, bivariate analyses, and breeding value evaluations are summarized in Table S9 (Supplementary Materials).

#### 2.3.2. Correlations, Confidence, and Credibility Intervals

Correlation estimates were calculated as:

$$r_{k}=\frac{{Cov}_{k}(y_{1},y_{2})}{\sqrt{{Var}_{k}(y_{1}){Var}_{k}(y_{2})}}$$

where $r_{k}$ represents additive genetic, permanent environmental or residual correlations.

Ninety-five percent confidence intervals (CI95%) and posterior credibility intervals (PCI95%) were calculated as $\hat{\theta }\pm 1.96{\hat{\sigma }}_{\hat{\theta }}$, using standard errors (SE) for CI95% and posterior standard deviations (PSD) for PCI95%, respectively. PCI95% intervals were included to facilitate comparison with REML-based confidence intervals. In addition, highest posterior density intervals (HPD95%), derived directly from the posterior samples, were computed and used as the primary Bayesian interval estimates.

### 2.4. Comparison of Full and Reduced Models

To assess the sensitivity of frequentist and Bayesian approaches to model complexity under limited sample size, full and reduced models were fitted for each trait. The full model included direct additive genetic effects and the doe’s permanent environmental effect, whereas the reduced model excluded the permanent environmental effect. Both models were independently fitted using AI-REML and MCMC-GS, keeping fixed or systematic effects and the animal model structure constant. Model comparisons were performed within each statistical approach.

#### 2.4.1. Model Fit and Parsimony

Under the REML framework, model fit was evaluated using restricted log-likelihood and the Akaike information criterion (AIC), along with numerical stability and convergence diagnostics. Under the Bayesian framework, model fit was assessed descriptively using the posterior mean deviance ($\bar{D}$), together with convergence diagnostics and posterior distribution characteristics.

#### 2.4.2. Comparison of Predicted Breeding Values

Breeding values (BV) were predicted for all 3481 animals represented in the pedigree. Agreement among BV was assessed using Spearman’s rank correlation, with particular emphasis on ranking stability. Additionally, the coincidence of animals within the top 10% and 20% of the population was evaluated. These thresholds were selected to represent high- and moderate-intensity selection scenarios and to assess the practical implications of method and model choice on the identification of elite animals.

## 3. Results

### 3.1. Variance Components

**Figure 1:**
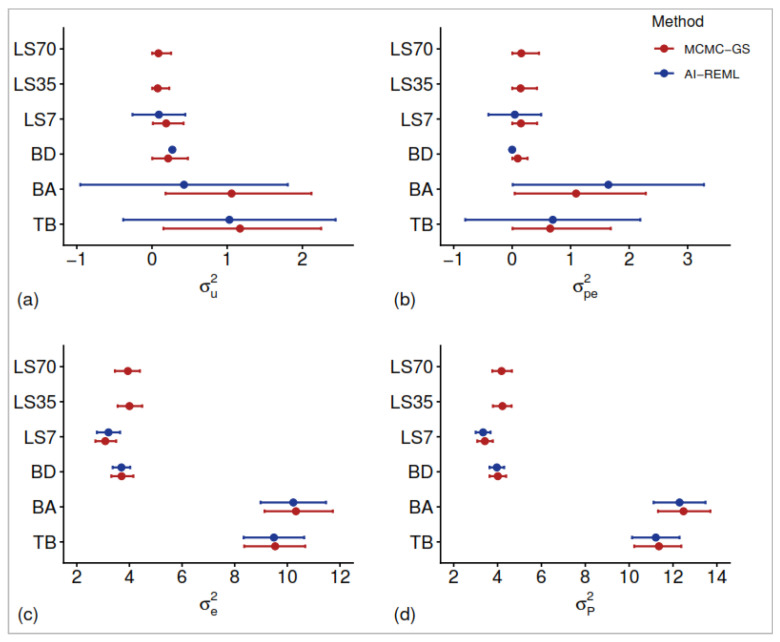
Estimates of additive genetic ($\sigma_{u}^{2}$), permanent environmental ($\sigma_{pe}^{2}$), residual ($\sigma_{e}^{2}$), and phenotypic variances ($\sigma_{P}^{2}$) for litter size traits in meat rabbits, using AI-REML and MCMC-GS (panels **a**–**d**, respectively). Points indicate point estimates, whereas horizontal bars represent 95% confidence intervals (AI-REML) and 95% highest posterior density intervals (MCMC-GS).

Estimates of variance components for litter size traits obtained using REML and MCMC-GS are shown in Figure 1. Numerical values for converged estimates and their SE or PSD are presented in Table S1 (Supplementary Materials). The EM-REML and AI-REML algorithms produced virtually identical point estimates for most traits, indicating that both converged to the same restricted maximum likelihood values. However, for LS35 and LS70, the EM-REML algorithm failed to satisfy the predefined convergence criteria. For this reason, subsequent comparisons were performed only between AI-REML and MCMC-GS. Under AI-REML, LS35 yielded variance component estimates that were effectively zero (σ^2^u = 9.07 × 10^−5^ and σ^2^pe = 5.78 × 10^−5^), indicating a boundary solution, whereas no stable solution was obtained for LS70. Comparisons involving these traits were therefore restricted to estimates considered valid under each analytical approach.

Overall, AI-REML and MCMC-GS yielded similar estimates of residual and total phenotypic variance. In contrast, relevant differences were observed in the magnitudes of additive genetic (σ^2^_u_) and permanent environmental variances (σ^2^_pe_), as well as in the associated uncertainties (SE or PSD). AI-REML yielded estimates close to zero, convergence problems, or relatively large standard errors for some traits. In contrast, MCMC-GS provided non-zero estimates and posterior distributions with well-defined intervals. The complete posterior distributions of σ^2^_u_ and σ^2^_pe_, together with those of h^2^ and repeatability, are presented in Figure S2 (Supplementary Materials). These distributions were generally unimodal, although posterior uncertainty increased for LS35 and LS70, reflecting the limited amount of information available for these traits. The supplementary figure allows direct assessment of posterior uncertainty and the concentration of posterior density near zero for all estimated parameters.

### 3.2. Genetic Parameters

**Figure 2:**
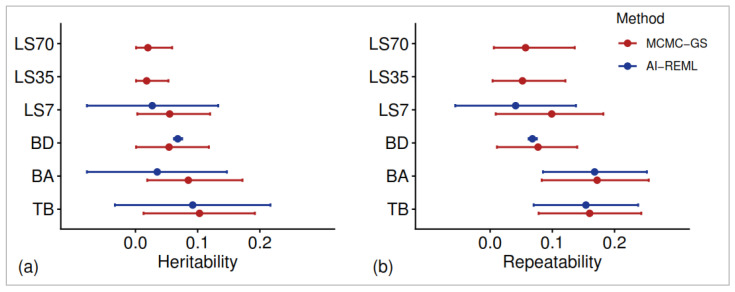
Heritability (**a**) and repeatability (**b**) estimates for litter size traits in meat rabbits, estimated using AI-REML and MCMC-GS. Points indicate point estimates, whereas horizontal bars represent 95% confidence intervals (AI-REML) and 95% highest posterior density intervals (MCMC-GS).

Figure 2 shows heritability and repeatability estimates, together with their confidence and credibility intervals (CI95% and HPD95%), obtained using AI-REML and MCMC-GS. The corresponding numerical values are reported in Table S2 (Supplementary Materials). Overall, h^2^ estimates were low for all traits, ranging from 0.03 to 0.09 with AI-REML and from 0.02 to 0.10 under the Bayesian approach. For most traits, except for BD, h^2^ point estimates were numerically higher under the Bayesian approach, although confidence and credibility intervals overlapped substantially for several traits. The total number of kits born showed the highest heritability with both methods.

Repeatability estimates were generally higher than heritability estimates and were slightly higher with MCMC-GS across all traits. Differences between approaches were smaller for traits recorded at birth and more pronounced for traits measured after birth. The highest repeatability was observed for BA and TB, with values ranging from 0.15 to 0.17 with both methods, whereas the lowest repeatability estimates were obtained for LS35 and LS70. In most cases, AI-REML yielded larger standard errors for h^2^ than the posterior standard deviations obtained with MCMC-GS. In contrast, the uncertainty associated with repeatability estimates was similar across methods.

### 3.3. Correlations

**Figure 3:**
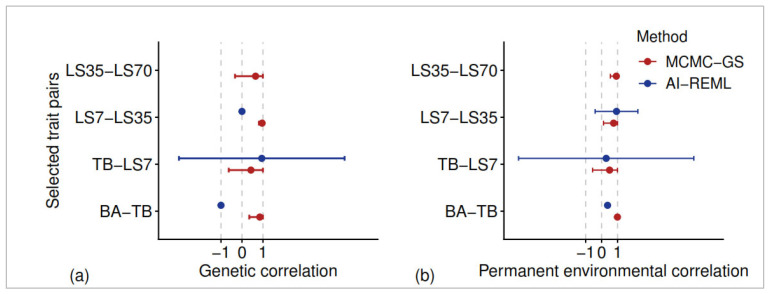
Estimates of genetic (**a**) and permanent environmental (**b**) correlations for selected pairs of litter size traits representing successive stages of litter development in meat rabbits, estimated using AI-REML and MCMC-GS. Points represent point estimates, whereas horizontal bars represent 95% confidence intervals (AI-REML) and 95% highest posterior density intervals (MCMC-GS). The vertical dashed lines indicate the theoretical lower and upper limits of the correlation coefficient (−1 and 1).

Genetic, permanent environmental, and residual correlations estimated using AI-REML and MCMC-GS are presented in Tables S3–S5 (Supplementary Materials). Figure 3 illustrates selected genetic and permanent environmental correlations for trait pairs representing consecutive stages of litter development, from birth (BA–TB) through pre-weaning (TB–LS7 and LS7–LS35) to post-weaning performance (LS35–LS70). Genetic correlations showed less consistent patterns between methodologies, with important differences in the magnitude of the estimates. In several cases, AI-REML produced extreme estimates, close to zero or one, accompanied by unusually small or very large standard errors. In contrast, MCMC-GS yielded estimates of intermediate magnitude, accompanied by relatively wide uncertainty intervals.

Permanent environmental correlations showed more consistent patterns across approaches, though notable differences in magnitude were observed. For example, the permanent environmental correlation between BA and TB was highest under MCMC-GS (0.99 ± 0.02), whereas it was much lower under AI-REML (0.37 ± 0.02). As observed for genetic correlations, AI-REML produced atypical estimates, close to zero or one, with standard errors either close to zero or larger than the point estimate, whereas MCMC-GS generally yielded estimates that were less extreme in magnitude. Residual correlations were generally similar between methodologies.

Genetic and permanent environmental correlations for TB–LS35, TB–LS70, and LS35–LS70 could not be estimated using AI-REML because the corresponding bivariate analyses failed to converge and were therefore excluded from further consideration. In addition, several of the remaining AI-REML correlation estimates were associated with boundary solutions in one or more variance components.

### 3.4. Full and Reduced Models

#### 3.4.1. Model Fit and Parsimony

**Figure 4:**
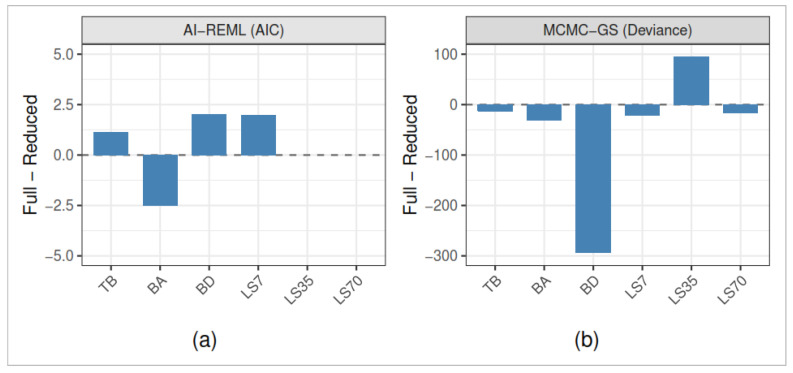
Differences in model fit (ΔAIC and Δ$\bar{D}$) between full and reduced models obtained using the frequentist (**a**) and Bayesian (**b**) approaches. Values calculated as Full—Reduced models. Negative values indicate better fit of the full model.

Differences in model fit metrics between the full and reduced models are summarized in Figure 4, which presents the differences in fit obtained using the Akaike Information Criterion under the frequentist approach and the posterior mean deviance under the Bayesian approach. In both cases, values correspond to the difference between the full and reduced models, so negative values indicate a better fit of the full model. The numerical values of the fit criteria are reported in the Supplementary Materials (Table S6). Most ΔAIC and Δ$\bar{D}$ values were relatively small, indicating similar fit between model structures for several traits.

Under AI-REML, differences in AIC between models were generally small (close to zero) across the traits for which model comparison was possible (TB, BA, BD, and LS7), indicating similar goodness-of-fit among model structures. Comparisons for LS35 and LS70 were not considered because the corresponding AI-REML analyses resulted in a boundary solution and lack of convergence, respectively.

Under the Bayesian approach, differences in $\bar{D}$ varied across traits. For BA, BD, TB, LS7, and LS70, the full model showed lower $\bar{D}$ values than the reduced model, indicating a better fit. In contrast, for LS35, the reduced model exhibited lower $\bar{D}$ values and was therefore favored over the full model (Figure 4). This result suggests that the amount of information available for this trait may have been insufficient to justify the additional complexity associated with the permanent environmental effect. Therefore, although inclusion of this effect may be biologically relevant, its statistical support can vary among traits depending on the balance between model complexity and available information.

#### 3.4.2. Additive Genetic Variance and Heritability

**Figure 5:**
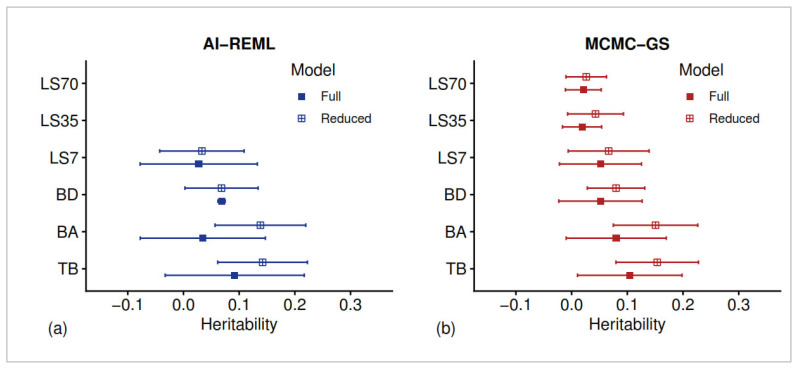
Heritability estimates, 95% confidence intervals (AI-REML) and 95% posterior credibility intervals (MCMC-GS) obtained using full and reduced models under the frequentist (**a**) and Bayesian (**b**) approaches.

Heritability estimates obtained using full and reduced models under AI-REML and MCMC-GS are shown in Figure 5, while detailed numerical values of variance components and genetic parameters are provided in the Supplementary Materials (Table S7). Overall, reduced models yielded higher estimates of σ^2^_u_ and h^2^ than full models across all traits and estimation approaches.

For traits recorded at birth (BA, BD, and TB), the differences between full and reduced models were moderate and consistent across approaches. In all cases, omission of the permanent environmental effect resulted in higher h^2^ estimates, although the magnitude of the increase varied among traits. Estimates from AI-REML and MCMC-GS showed similar patterns, although the Bayesian approach tended to yield slightly higher estimates.

For postnatal traits, differences between model structures were more pronounced. For LS7, both approaches showed a clear increase in h^2^ under the reduced model. Comparisons for LS35 and LS70 were not considered under AI-REML because the corresponding analyses resulted in a boundary solution and lack of convergence, respectively. In contrast, MCMC-GS produced positive estimates for both traits under the full and reduced model, with higher h^2^ estimates under the reduced model.

#### 3.4.3. Comparison of Predicted Breeding Values

**Figure 6:**
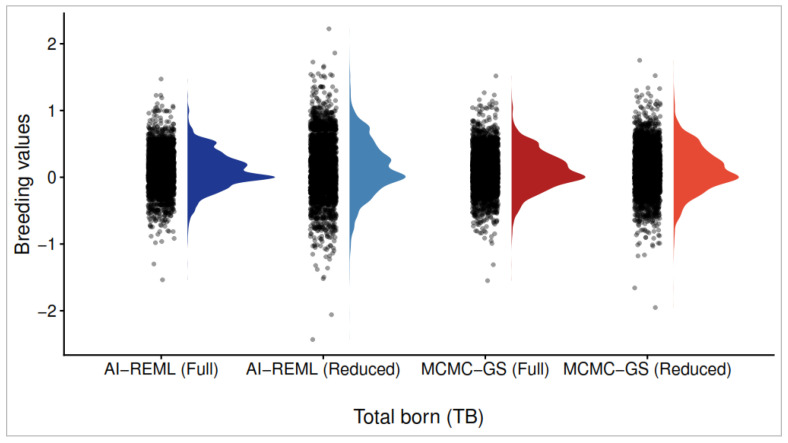
Distribution of predicted breeding values for the total born trait under full and reduced models using AI-REML and MCMC-GS.

Figure 6 shows the distribution of predicted breeding values for the TB trait obtained using full and reduced animal models under the frequentist (AI-REML) and Bayesian (MCMC-GS) approaches. Within both approaches, the full and reduced models produced very similar BV distributions. In both AI-REML and MCMC-GS, the reduced model showed a slightly greater dispersion of breeding values than the corresponding full model, although no appreciable shift in the central tendency of the distributions was observed.

The agreement between BV obtained from full and reduced models, evaluated using Spearman rank correlations and the percentage of coincident animals with the highest BV, is presented in Table 3. Under AI-REML, rank correlations between full and reduced models were virtually perfect (0.99–1.00), with overlap exceeding 87% and 94% for the top 10% and 20% of animals, respectively.

Under the Bayesian approach, correlations between BV predicted by full and reduced models were also high, ranging from 0.91 to 0.98. Similarly, percentages of coincidence in the top 10% and 20% of superior animals decreased relative to AI-REML but remained above 70% and 80%, in all cases, respectively.

The comparison between BV obtained with the reduced model under AI-REML and the full model under MCMC-GS also showed high agreement for TB, BA, BD, and LS7, with Spearman correlations between 0.97 and 0.99. LS70 comparisons involving AI-REML could not be performed because stable convergence was not achieved. Comparisons involving AI-REML were not performed for LS35 and LS70 because the corresponding full and reduced model analyses resulted in a boundary solution or failed to converge.

## 4. Discussion

### 4.1. Additive Genetic Variance and Heritability

The low estimates of additive genetic variance and heritability (h^2^ < 0.10) obtained in this study confirm that litter size traits in rabbits are strongly influenced by environmental, physiological, and management factors, and that additive genetic effects account for only a small proportion of the observed phenotypic variation. This pattern has been widely reported in previous studies about the genetics of reproductive traits in this species [6,7,20,23,24].

The highest h^2^ was observed for TB (0.09 and 0.10 with AI-REML and MCMC-GS, respectively), indicating that this trait may offer relatively greater potential for genetic improvement than the other litter size traits evaluated. Nevertheless, the low magnitude of the estimates suggests that the expected response to selection would remain limited. This finding is consistent with previous reports identifying litter size at birth as a trait exhibiting greater relative genetic variation than litter size measures recorded at later stages, which incorporate additional sources of environmental variation associated with survival and postnatal management [20,25].

### 4.2. Permanent Environmental Variance and Repeatability

Similarly, estimates of permanent environmental variance were low to moderate, with higher values for BA and TB, resulting in low to moderate repeatability estimates that exceeded the heritability estimates, as expected. These results are consistent with previous studies [7,26,27,28,29]. The decreasing trend in repeatability with increasing age of the kits may suggest that permanent environmental effects are relatively more important during early life stages, whereas specific environmental factors, such as competition among kits, resource availability, and post-weaning management, may become more influential at later stages. This pattern has been consistently reported in studies of reproductive and survival traits across species [30,31,32].

It is important to note that the data structure in this study imposed important limitations on the precise estimation of σ^2^_pe_. In particular, the high proportion of does with a single parity (40–48%) and the low proportion of does with five or more parities considerably reduced the amount of information available to statistically disentangle additive genetic effects from permanent environmental effects of the doe. This lack of repeated records per individual likely contributed to the high variability observed in σ^2^_pe_ estimates, especially under the frequentist approach. Accordingly, several studies agree that the accurate separation of additive genetic and permanent environmental variance components depends on the availability of multiple records per individual [8,9], and that study designs with few records per individual tend to yield less precise estimates of these components [10].

### 4.3. Genetic and Permanent Environmental Correlations

Genetic correlations estimated using MCMC-GS generally showed positive associations between BA and early litter size traits (TB, LS7, and LS35). In particular, the high r_g_ between LS7 and LS35 may suggest shared genetic influences between these traits, although the uncertainty associated with the estimates should be considered. Similar patterns have been reported in rabbits and other species, where early litter size measurements capture a substantial proportion of the genetic information associated with prolificacy [6]. In contrast, the low r_g_ estimates involving LS70 may indicate that litter size at later ages is influenced by additional factors not fully shared with traits recorded at earlier stages. However, given the uncertainty associated with several correlation estimates, these interpretations should be considered tentative.

A similar pattern was observed for permanent environmental correlations. Permanent environmental correlations estimated using MCMC-GS were generally positive among litter size traits recorded at consecutive stages of litter development. The highest estimate was observed between BA and TB (r_pe_ = 0.99 ± 0.02). This result may reflect the close biological and mathematical relationship between these traits, as BA represents the major component of TB and both traits are recorded at the same reproductive event.

### 4.4. Comparison Between Statistical Approaches

Variance component estimates obtained with AI-REML showed greater variability and inconsistencies than those obtained with MCMC-GS, particularly for σ^2^_u_ and σ^2^_pe_. In some cases, null estimates, large standard errors, or lack of convergence were observed, especially for LS70. This performance is consistent with the known sensitivity of the AI-REML algorithm to complex models, limited data structures, and (co)variance matrices that are close to positive semidefinite, which can lead to numerical conditioning problems and unreliable estimates [18].

In small populations, confounding among random effects and insufficient information to estimate specific variance components may cause AI-REML to converge to boundary solutions or fail to converge altogether. This phenomenon has been documented in both empirical studies and simulation analyses, where REML has been shown to produce unreliable estimates when information is scarce [33,34] or models are over-parameterized [18], reflecting a high sampling variance of the variance component estimators [35].

In contrast, the Bayesian approach based on Gibbs sampling yielded non-zero point estimates for all variance components. Although posterior standard deviations were relatively large, they explicitly reflect the uncertainty inherent in parameter estimation in small populations [14]. In large and well-structured populations, previous studies have reported high concordance between variance component estimates obtained by REML and Gibbs sampling, with minimal differences in both means and precision [36,37].

The heritability and repeatability estimates obtained using MCMC-GS were consistently higher than those obtained with AI-REML, in agreement with previous studies reporting differences in the performance of Bayesian and REML-based estimators when applied to small samples or complex data structures [38,39,40]. However, these differences do not necessarily imply greater accuracy, as the true parameter values are unknown. It has also been noted that Bayesian estimates may show slight upward bias for traits with low heritability and small population size, particularly when flat or weakly informative priors are used [11,40].

In terms of precision, AI-REML generally produced larger standard errors for h^2^ compared with the posterior standard deviations obtained using MCMC-GS, indicating greater uncertainty around the estimates obtained under the frequentist approach in this context. For repeatability, both approaches showed similar levels of precision, indicating that the estimation of residual and total phenotypic variance was less sensitive to the method used and to the amount of available information [14].

More generally, the wide confidence and credibility intervals observed for several variance components and genetic parameters reflect the limited amount of information available in the dataset. Consequently, estimates of genetic parameters and the resulting breeding values should be interpreted with appropriate caution. This limitation is particularly relevant for traits with low heritability and high uncertainty, where differences among animals may be more difficult to distinguish reliably. Under these conditions, differences observed between estimation approaches may reflect not only methodological characteristics but also the reduced identifiability of variance components arising from the available data structure.

Genetic and permanent environmental correlations estimated using AI-REML exhibited extreme values in some cases, close to zero or one, accompanied by unusually small or excessively large standard errors. Several bivariate analyses failed to converge, whereas many of the remaining estimates were associated with boundary solutions in one or more variance components. This performance suggests numerical instability associated with the complexity of the bivariate model and the limited information available to estimate covariances, as previously reported for multivariate models with sparse data structures or highly correlated parameters [18]. Under these conditions, correlations located near the limits of the parameter space should be interpreted cautiously, as they may reflect reduced parameter identifiability rather than strong biological relationships between traits.

In contrast, MCMC-GS produced moderate estimates of r_g_ and r_pe_, albeit with large posterior uncertainty. These large posterior standard deviations explicitly reflect the uncertainty associated with correlation estimation in small populations, particularly when non-informative priors are used, and inference relies primarily on the observed data [14]. In studies with larger datasets, similar r_g_ estimates have been reported using both approaches [40].

### 4.5. Comparison of Full and Reduced Models

The comparison between full models, including additive genetic and permanent environmental effects, and reduced models, excluding the permanent environmental effect, allowed an assessment of the stability of both statistical approaches to model complexity under limited sample size conditions. In the analysis of variance components for reproductive traits, the choice of model structure and statistical method is critical, as the inclusion of additional effects may improve biological soundness but also increase the risk of numerical instability when information is limited [18].

Model comparison results were generally trait-dependent. In several cases, full models showed improved fit and more detailed partitioning of phenotypic variance, whereas in others the differences between model structures were small or inconsistent across approaches. Under the Bayesian framework, the inclusion of the permanent environmental effect generally reduced the inflation of additive genetic variance observed in reduced models (Table S7). However, this effect also increased model complexity and exacerbated convergence and numerical instability problems in AI-REML, particularly for traits with fewer records or limited repetition per doe. In these cases, null estimates of σ^2^_u_ and σ^2^_pe_, large standard errors, or lack of convergence were observed, consistent with the findings of Misztal [18], who associated such performance with over-parameterized models or insufficient information to estimate certain variance components.

In contrast, under the Bayesian approach, full models yielded non-zero estimates of σ^2^_u_ and σ^2^_pe_, accompanied by posterior distributions that explicitly represented the associated uncertainty. This performance is expected, as Gibbs sampling does not rely on asymptotic normality assumptions and allows inference even in small populations, provided that posterior variability is properly interpreted [14,19]. However, posterior uncertainty was substantially greater for traits recorded at later stages, particularly LS35 and LS70, reflecting the limited amount of information available for these traits. Therefore, although posterior distributions could be obtained for all traits, variance component estimates associated with later litter size measurements should be interpreted with greater caution.

Previous studies have shown that, under these conditions, small differences in variance component estimates may translate into changes in predicted breeding values and animal ranking [18]. In the present study, rank correlations between breeding values obtained from the full and reduced models were consistently high. Likewise, the overlap among the top-ranked animals remained high across traits, although differences in ranking were observed, particularly under the Bayesian approach. In practical terms, these differences imply that a proportion of selection candidates may differ depending on the estimation method and model specification adopted. These results indicate that even when overall rank correlations are high, some re-ranking of elite animals may still occur, as previously highlighted in genetic evaluation studies [41].

With AI-REML, animal rankings were highly concordant across model structures, suggesting that omitting the permanent environmental effect had a limited impact on predicting genetic merit. This apparent stability may be explained by the tendency of the method to estimate σ^2^_pe_ near zero or with high uncertainty in small populations, effectively causing the full model to collapse into the reduced model and resulting in very similar breeding values [18,33].

Under MCMC-GS, breeding value rankings obtained from the full and reduced models also showed high agreement, although slight differences were observed for some traits. These differences likely reflect the redistribution of variance between additive genetic and permanent environmental components when the permanent environmental effect was included. Consequently, model specification may influence breeding value estimates and may lead to slight changes in the ranking of selection candidates, particularly when using Bayesian approaches that are more responsive to changes in model specification [17,18].

In this context, the stability of rankings obtained with AI-REML does not necessarily imply that the omitted effect is biologically irrelevant; rather, it may reflect the method’s limited ability to separate additional effects under restricted information adequately. In contrast, although the Bayesian approach produced slightly more variable rankings, this performance likely reflects a greater responsiveness of breeding value estimates to model specification under limited-information conditions, which is particularly relevant when selection decisions focus on a small subset of animals with the highest genetic merit.

### 4.6. Methodological and Practical Implications

Although litter size traits are discrete count variables, linear animal models remain the most widely used framework for routine genetic evaluation of prolificacy traits in rabbits and other livestock species [20,22,42], as they allow direct estimation and comparison of genetic parameters across studies under a unified modeling structure. In the present work, this framework was adopted to ensure consistency between frequentist (REML) and Bayesian (MCMC-GS) approaches and to focus on methodological comparison rather than distributional assumptions.

Overall, the results indicate that the performance of variance component estimation methods is closely related to the amount of available information and model complexity. When large and well-balanced datasets are available, asymptotic theory holds, and no substantial differences between REML and Bayesian approaches are expected [14]. However, in small populations, such as the one analyzed in this study, the Bayesian approach showed greater numerical stability and was less affected by convergence difficulties.

These findings reinforce the idea that model complexity should be carefully balanced against the amount of available data. Overly complex models may yield unstable estimates without providing meaningful practical improvements, whereas simpler models may adequately describe the trait’s biology and yield comparable breeding values with a lower risk of numerical instability [18].

From a practical perspective, both approaches remain useful in rabbit breeding programs. Bayesian methods may be particularly advantageous in small populations, datasets with limited pedigree information, or situations where convergence difficulties are frequently encountered. However, REML-based approaches remain attractive for routine genetic evaluations because of their lower computational requirements, widespread implementation, and generally comparable performance when sufficient information is available. Therefore, the choice between approaches should consider both the available data structure and the objectives of the evaluation.

## 5. Conclusions

Litter size traits exhibited a reduced additive genetic component, explaining less than 10% of the phenotypic variance in the studied populations. The positive genetic correlations observed among several evaluated traits suggest that selection focused on early reproductive stages may generate favorable correlated responses at later stages. The comparison between statistical approaches showed that under the limited-information conditions evaluated in this study, AI-REML was more prone to convergence difficulties and boundary solutions for some traits, whereas the Bayesian approach based on Gibbs sampling was less sensitive to these limitations. The differences observed in animal rankings suggest that both the inference method and model complexity can influence breeding value estimates and the identification of elite animals under limited-information conditions.

## Figures and Tables

**Table 1:** Data structure for litter size traits in meat rabbits.

Trait	Kindlings (*n*)	Does (*n*)	Kindlings Per Doe	Range of Kindlings	Does with Only One Kindling (%)
Total born	956	440	2.2	1 to 7	40.0
Born alive	956	440	2.2	1 to 7	40.0
Born dead	956	440	2.2	1 to 7	40.0
Litter size at 7 days	819	399	2.1	1 to 6	45.9
Litter size at 35 days	804	402	2.0	1 to 6	47.8
Litter size at 70 days	725	359	2.0	1 to 6	47.1

**Table 2:** Effects considered in the models used to estimate (co)variance components.

Trait	Breed	CG	TS	PS	u	pe
Total born	x	x	x	x	x	x
Born alive	x	x	x	x	x	x
Born dead	x	x	-	-	x	x
Litter size at 7 days	x	x	x	x	x	x
Litter size at 35 days	x	x	x	x	x	x
Litter size at 70 days	x	x	x	x	x	x

CG = contemporary group; TS = type of service; PS = physiological status of the doe at mating; u = animal additive genetic effect; pe = permanent environmental effect; x indicates that the effect was included in the model, whereas “–” indicates that the effect was not included.

**Table 3:** Rank correlations and the percentage of coincident animals with the highest predicted breeding values obtained from full and reduced models under frequentist (AI-REML) and Bayesian approaches (MCMC-GS).

Trait	Comparison (Full Vs. Reduced Models)	Spearman (r)	Top 10% Overlap (%)	Top 20% Overlap (%)
TB	AI-REML vs. AI-REML	1.00	87.1	97.3
	MCMC-GS vs. MCMC-GS	0.98	86.5	92.8
	MCMC-GS vs. AI-REML	0.99	87.9	93.4
BA	AI-REML vs. AI-REML	0.99	87.6	94.1
	MCMC-GS vs. MCMC-GS	0.98	87.4	90.1
	MCMC-GS vs. AI-REML	0.98	86.5	91.8
BD	AI-REML vs. AI-REML	1.00	100.0	99.6
	MCMC-GS vs. MCMC-GS	0.96	91.1	90.2
	MCMC-GS vs. AI-REML	0.98	93.1	92.4
LS7	AI-REML vs. AI-REML	1.00	97.1	99.9
	MCMC-GS vs. MCMC-GS	0.95	78.7	81.6
	MCMC-GS vs. AI-REML	0.97	85.6	84.1
LS35	MCMC-GS vs. MCMC-GS	0.94	69.8	80.0
LS70	MCMC-GS vs. MCMC-GS	0.91	81.6	83.9

TB = total number of kits born; BA = number of kits born alive; BD = number of kits born dead; LS7 = litter size at 7 days of age; LS35 = litter size at weaning (35 days); LS70 = litter size at 70 days of age; AI-REML = Average information restricted maximum likelihood; MCMC-GS = Markov chain Monte Carlo with Gibbs sampling. Comparisons involving AI-REML for LS35 and LS70 are not presented because the corresponding full-model analyses resulted in boundary solutions or failed to converge.

## Data Availability

The data presented in this study are available on reasonable request from the corresponding author.

## Supplementary Materials

The following supporting information can be downloaded at: https://www.mdpi.com/article/10.3390/ani16142192/s1; Table S1: Estimated variance components ± standard errors or posterior standard deviations for litter size traits in meat rabbits, obtained using EM-REML, AI-REML, and MCMC-GS; Table S2: Heritability (h^2^) and repeatability (re) estimates ± standard errors (AI-REML) and posterior standard deviations (MCMC-GS) for litter size traits in meat rabbits; Table S3: Genetic correlations ± standard errors or posterior standard deviations among litter size traits in meat rabbits, estimated using AI-REML (above the diagonal) and MCMC-GS (below the diagonal); Table S4: Permanent environmental correlations ± standard errors or posterior standard deviations among litter size traits in meat rabbits, estimated using AI-REML (above the diagonal) and MCMC-GS (below the diagonal); Table S5: Residual correlations ± standard errors or posterior standard deviations among litter size traits in meat rabbits, estimated using AI-REML (above the diagonal) and MCMC-GS (below the diagonal); Table S6: Model fit and parsimony criteria for full and reduced animal models fitted using AI-REML and MCMC-GS; Table S7: Variance components and genetic parameters ± standard errors or posterior standard deviations among litter size traits in meat rabbits obtained from full and reduced animal models using AI-REML and MCMC-GS; Table S8: Effective sample size (ESS) and Geweke convergence diagnostics for variance components estimated using MCMC-GS; Table S9: MCMC settings, burn-in periods, thinning intervals, and retained samples used for univariate analyses, bivariate analyses, and breeding value evaluations; Figure S1: Representative trace plots of additive genetic ($\sigma_{u}^{2}$), permanent environmental ($\sigma_{pe}^{2}$) and residual ($\sigma_{e}^{2}$) variance components obtained by MCMC-GS for BA and LS70; Figure S2: Posterior distributions of the additive genetic ($\sigma_{u}^{2}$) and permanent environmental ($\sigma_{pe}^{2}$) variance components, heritability ($h^{2}$) and repeatability (re) estimated using MCMC-GS for total born (TB), born alive (BA), born dead (BD), litter size at 7 days (LS7), litter size at 35 days (LS35), and litter size at 70 days (LS70).

## Abbreviations

The following abbreviations are used in this manuscript:

REML	Restricted maximum likelihood
MCMC-GS	Markov Chain Monte Carlo with Gibbs sampling
AI-REML	Average information restricted maximum likelihood
EM-REML	Expectation–maximization restricted maximum likelihood
BA	Born alive
BD	Born dead
TB	Total born
LS7	Litter size at 7 days of age
LS35	Litter size at 35 days of age
LS70	Litter size at 70 days of age
CI95%	Confidence intervals to 95% probability
PCI95%	Posterior credibility interval to 95% probability
HPD95%	Highest posterior density intervals
SE	Standard errors
PSD	Posterior standard deviations
AIC	Akaike information criterion
$\bar{D}$	Posterior mean deviance
BV	Predicted breeding values
σ^2^_u_	Additive genetic variance
σ^2^_pe_	Permanent environmental variance
$h^{2}$	Heritability
*re*	Repeatability
r_g_	Genetic correlation
r_pe_	Permanent environmental correlation
r_e_	Residual correlation

## References

[1] García M.L., Argente M.J. (2020). The genetic improvement in meat rabbits. Lagomorpha Characteristics.

[2] Cartuche L., Pascual M., Gómez E.A., Blasco A. (2014). Economic weights in rabbit meat production. World Rabbit Sci..

[3] Magaña-Valencia F., Jiménez C., Rodríguez-de Lara R., Ramírez-Valverde R., García-Muñiz J.G., Núñez-Domínguez R., Hidalgo J. (2025). Estimation of economic weights for growth, feed efficiency, health, and fertility traits in a breeding program for meat rabbits. Transl. Anim. Sci..

[4] Baselga M., García M., Sánchez J.P., Vicente J.S., Lavarda R. (2003). Analysis of reproductive traits in crosses among maternal lines of rabbits. Anim. Res..

[5] Drouilhet L., Gilbert H., Balmisse E., Ruesche J., Tircazes A., Larzul C., Garreau H. (2013). Genetic parameters for two selection criteria for feed efficiency in rabbits. J. Anim. Sci..

[6] Argente M.J., Calle E.W., García M.L., Blasco A. (2017). Correlated response in litter size components in rabbits selected for litter size variability. J. Anim. Breed. Genet..

[7] Badawy A.Y., Peiró R., Blasco A., Santacreu M.A. (2019). Correlated responses on litter size traits and survival traits after two-stage selection for ovulation rate and litter size in rabbits. Animal.

[8] Wilson A.J., Réale D., Clements M.N., Morrissey M.M., Postma E., Walling C.A., Kruuk L.E.B., Nussey D.H. (2010). An ecologist’s guide to the animal model. J. Anim. Ecol..

[9] Kruuk L.E.B., Hadfield J.D. (2007). How to separate genetic and environmental causes of similarity between relatives. J. Evol. Biol..

[10] Jablonszky M., Garamszegi L.Z. (2024). The effect of repeated measurements and within-individual variance on the estimation of heritability: A simulation study. Behav. Ecol. Sociobiol..

[11] Nishio M., Arakawa A. (2022). Performance of the No-U-Turn sampler in multi-trait variance component estimation using genomic data. Genet. Sel. Evol..

[12] Strandén I., Mäntysaari E.A., Lidauer M.H., Thompson R., Gao H. (2024). A computationally efficient algorithm to leverage average information REML for (co)variance component estimation in the genomic era. Genet. Sel. Evol..

[13] Psutka J.V., Psutka J. (2019). Sample size for maximum-likelihood estimates of Gaussian model depending on dimensionality of pattern space. Pattern Recognit..

[14] Sorensen D., Gianola D. (2002). Likelihood, Bayesian, and MCMC Methods in Quantitative Genetics.

[15] Rameez R., Jahageerdar S., Jayaraman J., Chanu T.I., Bangera R., Gilmour A. (2022). Evaluation of alternative methods for estimating the precision of REML-based estimates of variance components and heritability. Heredity.

[16] Misztal I., Tsuruta S., Lourenco D.A.L., Masuda Y., Aguilar I., Legarra A., Vitezica Z. (2018). Manual for BLUPF90 Family Programs.

[17] Mrode R.A., Pocrnic I., Mrode R.A., Pocrnic I. (2023). Use of Gibbs Sampling in Variance Component Estimation and Breeding Value Prediction. Linear Models for the Prediction of the Genetic Merit of Animals.

[18] Misztal I. (2008). Reliable computing in estimation of variance components. J. Anim. Breed. Genet..

[19] Gelman A., Carlin J.B., Stern H.S., Dunson D.B., Vehtari A., Rubin D.B. (2013). Bayesian Data Analysis.

[20] Lima N.H.S., Dsuze E.Z.L., Paiva D.A., Lima N.D.S., Gomes T.R., Paiva J.T. (2024). Meta-analysis of genetic parameters for economic traits in rabbit using a random-effects model. World Rabbit Sci..

[21] Belabbas R., Ilès I., Argente M.J., Ezzeroug R., Ainbaziz H., García M.L. (2023). Environmental and genetic factors affecting litter size components in rabbits. World Rabbit Sci..

[22] Ezzeroug R., Belabbas R., Argente M.J., Berbar A., Diss S., Boudjella Z., Talaziza D., Boudahdir N., García M.L. (2020). Genetic correlations for reproductive and growth traits in rabbits. Can. J. Anim. Sci..

[23] Antonini A.G., Corva S.G., Oyarzabal M.I. (2009). Selección por tamaño de camada en conejos para carne. BAG J. Basic Appl. Genet..

[24] Piles M., García M.L., Rafel O., Ramon J., Baselga M. (2006). Genetics of litter size in three maternal lines of rabbits: Repeatability versus multiple-trait models. J. Anim. Sci..

[25] Parra-Bracamonte G.M., Becerril-Martínez L., Sánchez-Dávila F., Esparza-Jiménez S., Albarrán-Portillo B., García-Martínez A., López-Villalobos N., Vázquez-Armijo J.F. (2025). Seasonal effects and heritability of litter size at birth and weaning in commercial rabbits in central Mexico (2015–2021). Vet. Sci..

[26] El-Din M.S. (2022). Genetic analysis of pre-weaning litter traits in V-line rabbits using a single-trait animal model. Arch. Agric. Sci. J..

[27] Behiry F.M., Shabaan H.M.A., Abdel-Kafy E.S.M. (2021). Characterization, genetic evaluation, and genetic trends for some reproductive traits. Egypt. Poult. Sci. J..

[28] El Nagar A.G.F. (2015). Genetic Analysis of Longevity in Specialized Lines of Rabbits. Ph.D. Thesis.

[29] Ziadi C., Mocé M.L., Laborda P., Blasco A., Santacreu M.A. (2013). Genetic selection for ovulation rate and litter size in rabbits: Estimation of genetic parameters and direct and correlated responses. J. Anim. Sci..

[30] Wilson A.J., Réale D. (2006). Ontogeny of additive and maternal genetic variance in wild populations. Am. Nat..

[31] Bell A.M., Hankison S.J., Laskowski K.L. (2009). The repeatability of behaviour: A meta-analysis. Anim. Behav..

[32] Sharif N., Ali A., Dawood M., Khan M.I.U.R., Do D.N. (2022). Environmental effects and genetic parameters for growth traits of Lohi sheep. Animals.

[33] Lin E.C., Berger P.J. (2001). Comparison of (co)variance component estimates in control populations of red flour beetle (*Tribolium castaneum*) using restricted maximum likelihood and Gibbs sampling. J. Anim. Breed. Genet..

[34] de Villemereuil P., Gimenez O., Doligez B. (2013). Comparing parent–offspring regression with frequentist and Bayesian animal models to estimate heritability in wild populations: A simulation study for Gaussian and binary traits. Methods Ecol. Evol..

[35] Visscher P.M., Goddard M.E. (2015). A general unified framework to assess the sampling variance of heritability estimates using pedigree or marker-based relationships. Genetics.

[36] Van Tassell C.P., Van Vleck L.D. (1996). Multiple-trait Gibbs sampler for animal models: Flexible programs for Bayesian and likelihood-based (co) variance component inference. J. Anim. Sci..

[37] Van Tassell C.P., Casella G., Pollak E.J. (1995). Effects of selection on estimates of variance components using Gibbs sampling and restricted maximum likelihood. J. Dairy Sci..

[38] Alijani S., Jasouri M., Pirany N., Daghigh Kia H. (2012). Estimation of variance components for some production traits of Iranian Holstein dairy cattle using Bayesian and AI-REML methods. Pak. Vet. J..

[39] Guan J., Wang W., Hu Y., Wang M., Tian T., Kong J. (2017). Estimation of genetic parameters for growth trait of turbot using Bayesian and REML approaches. Acta Oceanol. Sin..

[40] Waldmann P., Ericsson T. (2006). Comparison of REML and Gibbs sampling estimates of multi-trait genetic parameters in Scots pine. Theor. Appl. Genet..

[41] Ramírez-Valverde R., Hernández-Alvarez O.C., Núñez-Domínguez R., Ruíz-Flores A., García-Muñiz J.G. (2007). Univariate vs. multivariate analyses in genetic evaluation of growth traits in two cattle breeds. Agrociencia.

[42] Tinh N.H., Hao T.V., Bui A.P.N. (2021). Genetic parameters and litter trait trends of Danish pigs in South Vietnam. Anim. Biosci..

