# Supplementary material for: Genetic Parameter Estimation and Breeding Value Ranking for Litter Size Traits in Meat Rabbits: REML and Bayesian Inference Under Limited Data Conditions
Source: Animals (Basel). 2026 Jul 14;16(14):2192. doi: 10.3390/ani16142192 (PMC13406007; doi:10.3390/ani16142192)
Supplement: Supplementary file 1 [file animals-16-02192-s001.zip › animals-4334926-supplementary.pdf]

Supplementary Materials

Table S1. Estimated variance components ± standard errors or posterior standard deviations for litter size traits in meat rabbits, obtained using EM-REML, AI-REML, and MCMC-GS.

Method	Trait	σ_u^2	σ_{pe}^2	σ_e^2	σ_P^2
EM-REML	TB	1.030	0.695	9.491	11.216
AI-REML	TB	1.030 ± 0.720	0.695 ± 0.763	9.491 ± 0.586	11.216 ± 0.551
MCMC-GS	TB	1.171 ± 0.586	0.650 ± 0.570	9.538 ± 0.597	11.359 ± 0.559
EM-REML	BA	0.426	1.645	10.227	12.298
AI-REML	BA	0.426 ± 0.703	1.645 ± 0.833	10.227 ± 0.634	12.298 ± 0.603
MCMC-GS	BA	1.059 ± 0.549	1.093 ± 0.684	10.328 ± 0.674	12.480 ± 0.618
EM-REML	BD	0.271	0.00006	3.696	3.967
AI-REML	BD	0.271 ± 0.006	0.00005 ± 0.000	3.696 ± 0.169	3.967 ± 0.170
MCMC-GS	BD	0.215 ± 0.145	0.095 ± 0.079	3.703 ± 0.216	4.013 ± 0.190
EM-REML	LS7	0.092	0.045	3.205	3.341
AI-REML	LS7	0.092 ± 0.179	0.045 ± 0.230	3.205 ± 0.225	3.341 ± 0.173
MCMC-GS	LS7	0.189 ± 0.129	0.150 ± 0.139	3.083 ± 0.205	3.422 ± 0.179
MCMC-GS	LS35	0.076 ± 0.072	0.144 ± 0.139	4.002 ± 0.244	4.223 ± 0.218
MCMC-GS	LS70	0.086 ± 0.078	0.156 ± 0.147	3.941 ± 0.241	4.182 ± 0.230

TB = total number of kits born; BA = number of kits born alive; BD = number of kits born dead; LS7 = litter size at 7 days of age; LS35 = litter size at weaning; LS70 = litter size at 70 days of age; AI-REML = restricted maximum likelihood using the average information algorithm; EM-REML = restricted maximum likelihood using the expectation maximization algorithm; MCMC-GS = Markov chain Monte Carlo using Gibbs sampling. σ_u^2 = additive genetic variance; σ_{pe}^2 = permanent environmental variance; σ_e^2 = residual variance; σ_p^2 = phenotypic variance. EM-REML and AI-REML estimates for LS35 and LS70 are not presented because the corresponding analyses resulted in boundary solutions or failed to converge.

Table S2. Heritability (h^2) and repeatability (re) estimates ± standard errors (AI-REML) and posterior standard deviations (MCMC-GS) for litter size traits in meat rabbits.

Trait	AI-REML		MCMC-GS	
	h^2	re	h^2	re
TB	0.092 ± 0.064	0.154 ± 0.043	0.103 ± 0.050	0.160 ± 0.042
BA	0.035 ± 0.057	0.168 ± 0.043	0.085 ± 0.043	0.172 ± 0.044
BD	0.068 ± 0.003	0.068 ± 0.003	0.054 ± 0.036	0.077 ± 0.035
LS7	0.027 ± 0.054	0.041 ± 0.049	0.055 ± 0.037	0.099 ± 0.049
LS35	-	-	0.018 ± 0.017	0.052 ± 0.037
LS70	-	-	0.020 ± 0.018	0.057 ± 0.039

TB = total number of kits born; BA = number of kits born alive; BD = number of kits born dead; LS7 = litter size at 7 days of age; LS35 = litter size at weaning; LS70 = litter size at 70 days of age; AI-REML = restricted maximum likelihood using the average information algorithm; MCMC-GS = Markov chain Monte Carlo using Gibbs sampling. AI-REML estimates for LS35 and LS70 are not presented because the corresponding analyses resulted in boundary solutions or failed to converge.

Table S3. Genetic correlations ± standard errors or posterior standard deviations among litter size traits in meat rabbits, estimated using AI-REML (above the diagonal) and MCMC-GS (below the diagonal).

Trait	BA	BD	TB	LS7	LS35	LS70
BA		0.452 ± 0.032	-1 ± 0	0 ± 0	0 ± 0	0 ± 0
BD	-0.047 ± 0.473		0 ± 0	-0.775 ± 1.165	-0.806 ± 0.006	-0.996 ± 0.012
TB	0.850 ± 0.228	0.508 ± 0.467		0.944 ± 2.014	-	-
LS7	0.795 ± 0.249	-0.791 ± 0.294	0.428 ± 0.524		0 ± 0	0 ± 0
LS35	0.804 ± 0.310	-0.864 ± 0.213	0.678 ± 0.380	0.960 ± 0.071		-
LS70	0.328 ± 0.470	-0.330 ± 0.531	-0.006 ± 0.521	0.287 ± 0.738	0.648 ± 0.409	

TB = total number of kits born; BA = number of kits born alive; BD = number of kits born dead; LS7 = litter size at 7 days of age; LS35 = litter size at weaning; LS70 = litter size at 70 days of age; AI-REML = restricted maximum likelihood using the average information algorithm; MCMC-GS = Markov chain Monte Carlo using Gibbs sampling. AI-REML correlations for TB–LS35, TB–LS70, and LS35–LS70 are not presented because the corresponding bivariate analyses failed to converge.

Table S4. Permanent environmental correlations ± standard errors or posterior standard deviations among litter size traits in meat rabbits, estimated using AI-REML (above the diagonal) and MCMC-GS (below the diagonal).

Trait	BA	BD	TB	LS7	LS35	LS70
BA		0.510 ± 0.072	0.373 ± 0.024	-0.667 ± 0.709	-0.686 ± 0.058	0.648 ± 0.065
BD	-0.830 ± 0.257		-0.017 ± 0.017	-0.998 ± 1.093	-0.106 ± 0.000	0.323 ± 0.004
TB	0.991 ± 0.016	-0.624 ± 0.525		0.286 ± 2.804	-	-
LS7	0.704 ± 0.288	-0.741 ± 0.429	0.500 ± 0.469		0.933 ± 0.684	-0.069 ± 0.211
LS35	0.207 ± 0.531	-0.253 ± 0.614	0.119 ± 0.566	0.750 ± 0.335		-
LS70	0.332 ± 0.603	-0.853 ± 0.296	0.412 ± 0.568	0.661 ± 0.318	0.922 ± 0.158	

TB = total number of kits born; BA = number of kits born alive; BD = number of kits born dead; LS7 = litter size at 7 days of age; LS35 = litter size at weaning; LS70 = litter size at 70 days of age; AI-REML = restricted maximum likelihood using the average information algorithm; MCMC-GS = Markov chain Monte Carlo using Gibbs sampling. AI-REML correlations for TB–LS35, TB–LS70, and LS35–LS70 are not presented because the corresponding bivariate analyses failed to converge.

Table S5. Residual correlations ± standard errors or posterior standard deviations among litter size traits in meat rabbits, estimated using AI-REML (above the diagonal) and MCMC-GS (below the diagonal).

Trait	BA	BD	TB	LS7	LS35	LS70
BA		-0.374 ± 0.039	0.752 ± 0.013	0.277 ± 0.049	0.207 ± 0.052	0.198 ± 0.054
BD	-0.356 ± 0.037		0.249 ± 0.250	-0.230 ± 0.042	-0.233 ± 0.031	-0.206 ± 0.031
TB	0.808 ± 0.014	0.249 ± 0.04		0.166 ± 0.047	-	-
LS7	0.280 ± 0.044	-0.212 ± 0.044	0.169 ± 0.047		0.832 ± 0.016	0.573 ± 0.039
LS35	0.220 ± 0.046	-0.224 ± 0.043	0.092 ± 0.048	0.806 ± 0.015		-
LS70	0.200 ± 0.049	-0.192 ± 0.047	0.099 ± 0.051	0.557 ± 0.032	0.720 ± 0.022	

TB = total number of kits born; BA = number of kits born alive; BD = number of kits born dead; LS7 = litter size at 7 days of age; LS35 = litter size at weaning; LS70 = litter size at 70 days of age; AI-REML = restricted maximum likelihood using the average information algorithm; MCMC-GS = Markov chain Monte Carlo using Gibbs sampling. AI-REML correlations for TB–LS35, TB–LS70, and LS35–LS70 are not presented because the corresponding bivariate analyses failed to converge.

Table S6. Model fit and parsimony criteria for full and reduced animal models fitted using AI-REML and MCMC-GS.

Trait	Method	Model	-2logLik	AIC	Deviance ($\overline{D}$)
TB	AI-REML	Full	4832.550	4838.550	-
	AI-REML	Reduced	4833.421	4837.421	-
	MCMC-GS	Full	-	-	4862.879
	MCMC-GS	Reduced	-	-	4875.311
BA	AI-REML	Full	4914.065	4920.065	-
	AI-REML	Reduced	4918.570	4922.570	-
	MCMC-GS	Full	-	-	4939.427
	MCMC-GS	Reduced	-	-	4969.622
BD	AI-REML	Full	3926.615	3932.615	-
	AI-REML	Reduced	3926.614	3930.614	-
	MCMC-GS	Full	-	-	3664.161
	MCMC-GS	Reduced	-	-	3957.734
LS7	AI-REML	Full	3219.022	3225.022	-
	AI-REML	Reduced	3219.056	3223.056	-
	MCMC-GS	Full	-	-	3242.995
	MCMC-GS	Reduced	-	-	3264.758
LS35	MCMC-GS	Full	-	-	3494.061
	MCMC-GS	Reduced	-	-	3399.497
LS70	MCMC-GS	Full	-	-	3053.114
	MCMC-GS	Reduced	-	-	3069.517

logLik = restricted log-likelihood; AIC = Akaike Information Criterion; Deviance ($\overline{D}$) = posterior mean deviance for Bayesian analyses. TB = total number of kits born; BA = number of kits born alive; BD = number of kits born dead; LS7 = litter size at 7 days of age; LS35 = litter size at weaning; LS70 = litter size at 70 days of age; AI-REML = restricted maximum likelihood using the average information algorithm; MCMC-GS = Markov chain Monte Carlo using Gibbs sampling. AI-REML full and reduced model estimates for LS35 and LS70 are not presented because the corresponding analyses resulted in boundary solutions or failed to converge.

Table S7. Variance components and genetic parameters ± standard errors or posterior standard deviations among litter size traits in meat rabbits obtained from full and reduced animal models using AI-REML and MCMC-GS.

Trait	Method	Model	σ^2_u	σ^2_e	h^2	re
TB	AI-REML	Full	1.030 ± 0.720	9.491 ± 0.586	0.092 ± 0.064	0.154 ± 0.043
	AI-REML	Reduced	1.597 ± 0.487	9.651 ± 0.567	0.142 ± 0.041	- -
	MCMC-GS	Full	1.192 ± 0.562	9.520 ± 0.600	0.104 ± 0.048	0.163 ± 0.043
	MCMC-GS	Reduced	1.757 ± 0.470	9.638 ± 0.563	0.154 ± 0.038	- -
BA	AI-REML	Full	0.426 ± 0.703	10.227 ± 0.634	0.035 ± 0.057	0.168 ± 0.043
	AI-REML	Reduced	1.707 ± 0.539	10.655 ± 0.629	0.138 ± 0.042	- -
	MCMC-GS	Full	1.004 ± 0.584	10.314 ± 0.664	0.080 ± 0.046	0.175 ± 0.044
	MCMC-GS	Reduced	1.895 ± 0.528	10.638 ± 0.621	0.151 ± 0.039	- -
BD	AI-REML	Full	0.271 ± 0.006	3.696 ± 0.170	0.068 ± 0.003	0.068 ± 0.003
	AI-REML	Reduced	0.271 ± 0.135	3.697 ± 0.206	0.068 ± 0.034	- -
	MCMC-GS	Full	0.208 ± 0.155	3.708 ± 0.223	0.052 ± 0.038	0.074 ± 0.037
	MCMC-GS	Reduced	0.321 ± 0.109	3.691 ± 0.197	0.080 ± 0.026	- -
LS7	AI-REML	Full	0.092 ± 0.179	3.205 ± 0.225	0.027 ± 0.054	0.041 ± 0.050
	AI-REML	Reduced	0.111 ± 0.110	3.230 ± 0.202	0.033 ± 0.039	- -
	MCMC-GS	Full	0.178 ± 0.133	3.088 ± 0.207	0.052 ± 0.038	0.097 ± 0.050
	MCMC-GS	Reduced	0.227 ± 0.130	3.169 ± 0.195	0.067 ± 0.037	- -
LS35	MCMC-GS	Full	0.079 ± 0.077	4.011 ± 0.231	0.019 ± 0.018	0.050 ± 0.035
	MCMC-GS	Reduced	0.182 ± 0.110	4.035 ± 0.233	0.043 ± 0.026	- -
LS70	MCMC-GS	Full	0.087 ± 0.069	3.961 ± 0.238	0.021 ± 0.016	0.052 ± 0.030
	MCMC-GS	Reduced	0.109 ± 0.079	4.056 ± 0.236	0.026 ± 0.019	- -

TB = total number of kits born; BA = number of kits born alive; BD = number of kits born dead; LS7 = litter size at 7 days of age; LS35 = litter size at weaning; LS70 = litter size at 70 days of age; AI-REML = restricted maximum likelihood using the average information algorithm; MCMC-GS = Markov chain Monte Carlo using Gibbs sampling; σ^2_u = additive genetic variance; σ^2_e = residual variance; h^2 = heritability; re = repeatability. AI-REML full and reduced model estimates for LS35 and LS70 are not presented because the corresponding analyses resulted in boundary solutions or failed to converge.

Table S8. Effective sample size (ESS) and Geweke convergence diagnostics for variance components estimated using MCMC-GS.

Trait	σ_u^2		σ_{pe}^2		σ_e^2	
	ESS	Geweke	ESS	Geweke	ESS	Geweke
TB	40.6	0.27	18.4	-0.46	802	0.14
BA	37.6	0.2	32.6	-0.31	183.4	0.15
BD	33.5	-0.31	69.9	-0.09	626.4	0.14
LS7	32.3	-0.19	49.3	-0.04	209.5	0.13
LS35	30.9	-0.46	57.7	-0.04	276.1	0.16
LS70	14.8	-0.57	60.7	0.12	379.0	0.09

TB = total number of kits born; BA = number of kits born alive; BD = number of kits born dead; LS7 = litter size at 7 days of age; LS35 = litter size at weaning; LS70 = litter size at 70 days of age; σ^2_u = additive genetic variance; σ_{pe}^2 = permanent environmental variance; σ^2_e = residual variance; ESS = effective sample size; Geweke = Geweke convergence diagnostic (Z statistic).

Table S9. MCMC settings, burn-in periods, thinning intervals, and retained samples used for univariate analyses, bivariate analyses, and breeding value evaluations.

Trait	Samples	Burn_in	Retained samples (number)
Variance components	200,000	10,000	3800
Breeding values	50,000	10,000	800
Correlations			
BA-BD	200,000	5,000	3,900
BA-TB	100,000	5,000	1,900
BA-LS7	100,000	500	1,990
BA-LS35	100,000	1,000	1,980
BA-LS70	100,000	12,000	1,760
BD-TB	100,000	7,500	1,850
BD-LS7	100,000	5,000	1,900
BD-LS35	100,000	7,500	1,850
BD-LS70	100,000	6,500	1,870
TB-LS7	100,000	2,500	1,950
TB-LS35	100,000	7,500	1,850
TB-LS70	100,000	3,750	1,925
LS7-LS35	100,000	4,000	1,920
LS7-LS70	100,000	2,500	1,950
LS35-LS70	100,000	5,000	1,900

TB = total number of kits born; BA = number of kits born alive; BD = number of kits born dead; LS7 = litter size at 7 days of age; LS35 = litter size at weaning; LS70 = litter size at 70 days of age.

For bivariate analyses, burn-in lengths were determined individually for each trait combination based on visual inspection of trace plots and convergence diagnostics. Differences among analyses reflected variation in chain mixing behavior and the number of iterations required to reach stationarity.

Figure S1. Representative trace plots of additive genetic (σ_u^2), permanent environmental (σ_{pe}^2) and residual (σ_e^2) variance components obtained by MCMC-GS for BA and LS70.

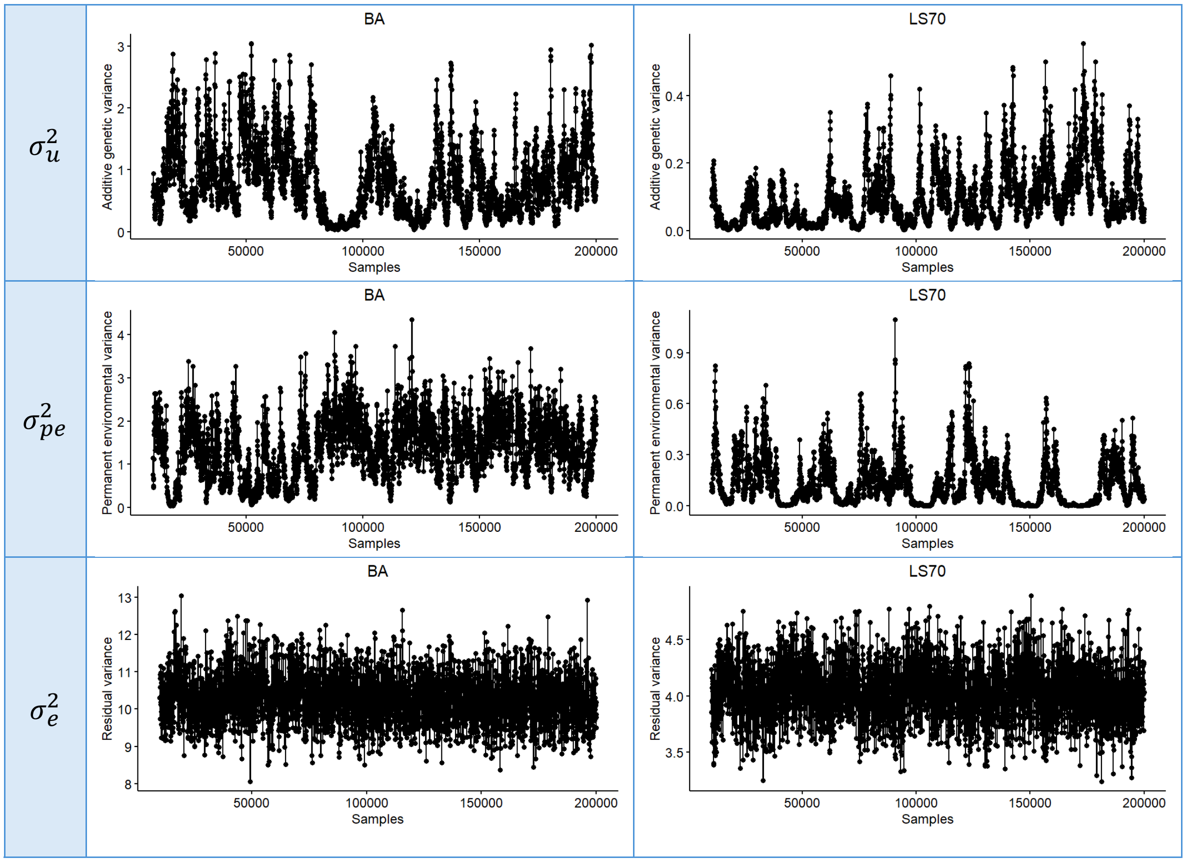

Trace plots correspond to additive genetic variance (σ_u^2), permanent environmental variance (σ_{pe}^2) and residual variance (σ_e^2). BA and LS70 were selected to illustrate chain behavior for traits exhibiting relatively low and high levels of posterior uncertainty, respectively.

Figure S2. Posterior distributions of the additive genetic (σ_u^2) and permanent environmental (σ_{pe}^2) variance components, heritability (h^2) and repeatability (re) estimated using MCMC-GS for total born (TB), born alive (BA), born dead (BD), litter size at 7 days (LS7), litter size at 35 days (LS35), and litter size at 70 days (LS70).

Trait	Additive genetic variance (σ_u^2)	Permanent environmental variance (σ_{pe}^2)	Heritability (h²)	Repeatability (re)
Born alive (BA)	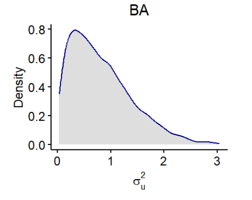	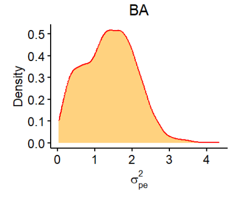	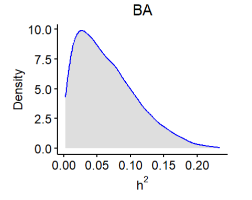	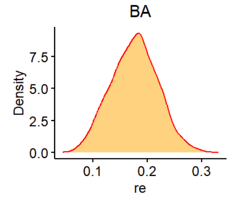
Born dead (BD)	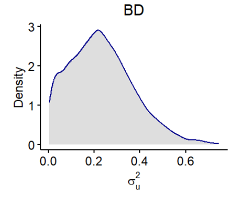	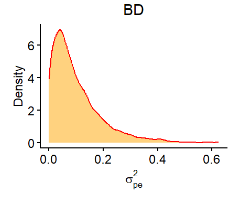	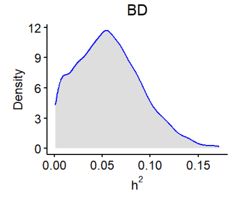	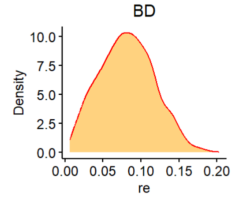
Total born (TB)	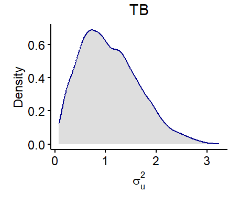	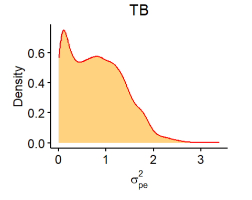	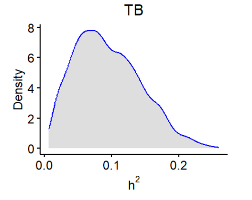	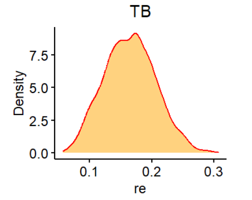
Litte size at 7 days (LS7)	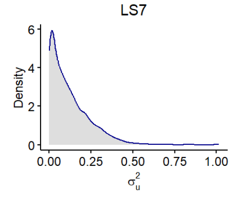	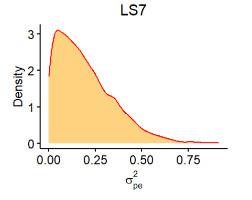	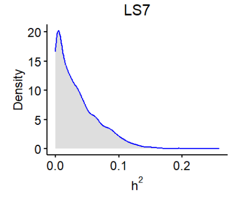	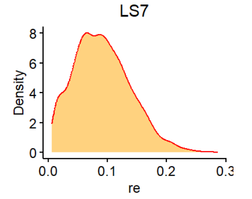
Litte size at 35 days (LS35)	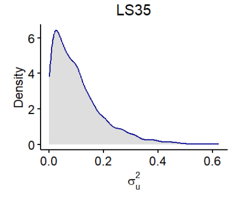	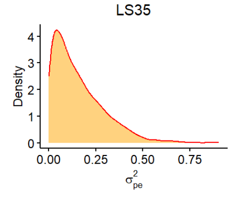	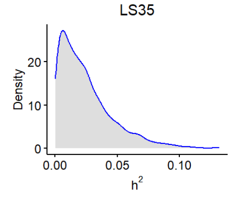	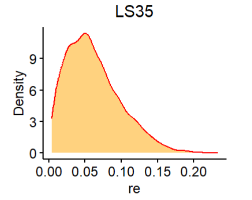
Litte size at 70 days (LS70)	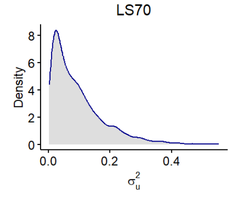	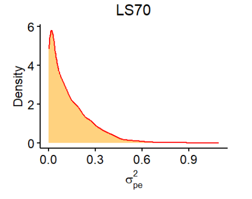	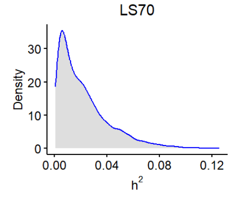	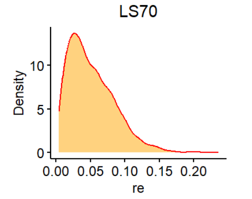
